# Fatal co-infection with leptospirosis and dengue in a Sri Lankan male

**DOI:** 10.1186/s13104-015-1321-7

**Published:** 2015-08-13

**Authors:** Aruna Wijesinghe, Nanthini Gnanapragash, Gayan Ranasinghe, Murugapillai K Ragunathan

**Affiliations:** National Hospital of Sri Lanka, Colombo, Sri Lanka

**Keywords:** Dengue fever, Leptospirosis, Co-infection

## Abstract

**Background:**

Leptospirosis and dengue are endemic in countries with subtropical or tropical climates and have epidemic potential. The incidence of both these diseases peaks during monsoons and both diseases present with similar clinical manifestations making differentiation of leptospirosis from dengue difficult. It is important to distinguish leptospirosis from dengue as early antibiotic therapy in leptospirosis leads to a favourable outcome, while dengue has no specific treatment, yet early recognition is vital for close monitoring and careful fluid management. Despite the high prevalence of both these infections, co-infection of leptospirosis and dengue has not been reported previously in Sri Lanka. We present the first case of co-infection with leptospirosis and dengue in a Sri Lankan male.

**Case presentation:**

A 52 year old previously healthy Sri Lankan male was admitted to our facility with a history of fever for 4 days associated with headache, generalized myalgia, reduced urine output. On examination, he was rational, hypotensive, tacycardic, tacypneic and he did not have clinical evidence of fluid leakage or pneumonitis. His serology showed high titre of dengue IgG and IgM and rising titre of leptospirosis antibody. His course of illness was complicated with septic shock, acute renal failure, acute respiratory distress syndrome and disseminated intravascular coagulation and he succumbed to his illness on the eighth day of admission.

**Conclusion:**

In areas where both leptospirosis and dengue are endemic, both infections should be include in the differential diagnosis when evaluating patients with acute febrile illness and should consider the possibility of co-infection. Leptospirosis, being a condition having definitive antibiotic therapy, should always be ruled out even if the patient is positive for dengue serology in regions endemic to both these diseases as early initiation of antibiotic therapy can reduce mortality significantly.

## Background

Leptospirosis, a zoonotic infection and dengue, an arthropod born viral infection, are endemic in countries with humid subtropical or tropical climates and have epidemic potential [1]. Both these infections have emerged as major public health problem in Sri Lanka in recent years [2]. Recently, Sri Lanka has experienced a string of widespread dengue epidemics annually which has now become a hyperendemic with frequent reports of dengue hemorrhagic fever (DHF) and dengue shock syndrome (DSS) [3].

The incidence of both these diseases peaks during monsoons and both diseases present with similar clinical manifestations making differentiation of leptospirosis from dengue difficult [4]. It is important to distinguish leptospirosis from dengue as early antibiotic therapy in leptospirosis leads to a favourable outcome, while dengue has no specific treatment, yet early recognition is vital for close monitoring and careful fluid management [5, 6].

Despite the high prevalence of both these infections, co-infection of leptospirosis and dengue has not been reported previously in Sri Lanka. We present the first case of co-infection with leptospirosis and dengue in a Sri Lankan male.

## Case presentation

A 52 year old previously healthy Sri Lankan male was admitted to our facility with a history of fever for 4 days associated with headache, abdominal pain, generalized myalgia and reduced urine output. On admission he was febrile, rational and nonicteric, with a blood pressure of 90/60 mmHg, pulse of 118 beats/min, breathing frequency of 22/min and oxygen saturation of 97 % on room air. The liver was tender and palpable 3 cm below the right costal margin. He did not have clinical evidence of pneumonitis, pleural effusion, ascites. Laboratory investigations on admission showed thrombocytopenia with normal white cell count and increased levels of blood urea, serum creatinine, liver transaminases, prothombin time, creatinine phosphokinase and trophonin I. Both IgG and IgM dengue antibody tests were positive indicating recent dengue infection. Serological tests for leptospirosis (microscopic agglutination test) was negative. The investigation results are summarized in Table 1. Despite adequate fluid resuscitation, the patient remained hypotensive and he was started on inotropes and vasopressors. He was started on ceftriaxone and transferred to an intensive care unit where haemodialysis was started due to worsening acidaemia and fluid overload. On the second day, he developed haemoptysis and chest X ray showed evidence of diffuse bilateral alveolar haemorrhages. Arterial blood gas analysis showed type 1 respiratory failure. As the patient was in severe respiratory distress, he was placed on mechanical ventilation. Later his condition was further complicated by disseminated intravascular coagulation. Leptospirosis serology was repeated on day 7 and it showed a rising titre of 3,600, indicating recent leptospirosis infection. Despite aggressive management of septic shock, oliguric acute renal failure, acute respiratory distress syndrome and disseminated intravascular coagulation, the patient succumbed to his illness on the eighth day of admission.

**Table 1:** Laboratory investigations of case presentation

Days from admission	Day 1	Day 3	Day 5	Day 8	Reference value
Haemoglobin (g/L)	11.4	11.3	7.3	9.1	13–16
Haematocrit (%)	32.8	32.6	24.6	28.3	40–50
Platelets (×10^9^/L)	23	9	36	121	150–450
White blood cells (×10^9^/L)	8.7	16.8	23.1	28.6	4,000–10,000
CPK (u/L)	1,180	1,790	809	–	25–174
Serum creatinine (µmol/L)	288	405	378	426	0.6–1.2
Blood urea (mmol/L)		28.9	32.8	34.7	2.9–8.2
Sodium (mmol/L)	133	137	136	142	135–148
Potassium (mmol/L)	4.3	3.9	4.8	5.4	3.5–5.1
AST (u/L)	48	151	109	126	10–35
ALT (u/L)	49	74	88	92	10–40
Serum bilirubin (µmol/L)	84	230	690	837	5.1–22
INR	1.26	1.8	2.1	3.2	1–1.3
APTT	58	69	87.9	116	28–42

## Discussion

Leptospirosis and dengue are increasingly being recognized as a cause of acute febrile illness in tropics and subtropics, putting almost half of the world’s population at risk. Sri Lanka, with a population of 25 million, represents an endemic region for both leptospirosis and dengue infections due to the geographic and climatic aspects of the country and the socioeconomic characteristics of its population.

Dengue fever is currently the most important mosquito-borne viral infection of public health significance in Sri Lanka, with thousands of patients acquiring the infection each year [3]. Dengue fever is caused by any of the four dengue virus serotypes (DEN 1–4) which are closely related. Dengue virus serotypes 2 and 3 were the predominant circulating serotypes in Sri Lanka until 2009. However, since 2010, dengue serotype 1 has become the predominant serotype in Sri Lanka accounting for more than 95 % of dengue infections [3]. Leptospirosis is a zoonotic disease which is endemic in Sri Lanka. The reported annual incidence of leptospirosis in Sri Lanka is 5.4 cases/100,000 persons, the sixth highest incidence worldwide [7]. However, incidence rates are imprecise estimates because leptospirosis is easily confused with undifferentiated fever of other causes, and few cases are laboratory confirmed [8]. There have been a tremendous increase in the number of cases reported in the recent years, with 7,421 cases (incidence rate 36.7/100,000 population) and 207 deaths (case fatality ate 2.8 %) reported in 2008 [9].

Cases of dengue and leptospirosis were seen throughout the year with incidence of both disease peak during the monsoon leading to concurrent epidemics. The clinical manifestations of leptospirosis and dengue range from a mild self-limiting febrile illness to a severe and potentially fatal illness characterized by thrombocytopenia, bleeding, hepatitis with cholestatic jaundice, myositis and renal failure. The vast overlapping spectrum of symptomatic manifestations of dengue and leptospirosis makes the clinical diagnosis challenging for treating physicians when acute co-infection is present.

An Indian study revealed that 1.7 % of patients presented with acute febrile illness have co-infection with leptospirosis and dengue [4]. As Sri Lanka and India share common geographic, climatic and socio-demographic characteristics, a similar incidence of co-infection with leptospirosis and dengue can be expected in Sri Lanka as well. The fact that the co-infection with leptospirosis and dengue has not been previously reported in Sri Lanka is most probably due to under-diagnosis and under-reporting rather than the rarity of its occurrence.

Even though co-infection with leptospirosis and dengue carries a high mortality rate, early administration of appropriate antibiotics is important as it has been shown to reduce the duration and severity of leptospirosis [5]. Therefore, clinicians in areas where both leptospirosis and dengue are endemic should include both infections in the differential diagnosis when evaluating patients with acute febrile illness and should consider the possibility of co-infection.

## Conclusion

In areas where both leptospirosis and dengue are endemic, both infections should be include in the differential diagnosis when evaluating patients with acute febrile illness and should consider the possibility of co-infection. Leptospirosis, being a condition having definitive antibiotic therapy, should always be ruled out even if the patient is positive for dengue serology in regions endemic to both these diseases as early initiation of antibiotic therapy can reduce mortality significantly.

## Consent

Written informed consent was obtained from the patient’s next of kin for publication of this case presentation. A copy of the written consent is available for review by the Editor of this journal.

## References

[1] Pan American Health Organization (2000) Case definitions: dengue and leptospirosis. Epidemiol Bull. Available at: http://www.paho.org/english/sha/be_v21n2-cases.htm. Accessed 7 July 2015

[2] Epidemology Unit (2012) Weekly epidemilogical Report. 39:1–3. Available at: http://www.epid.gov.lk/web/attachments/article/188/Vol%2039%20NO%2001%20English.pdf. Accessed 7 July 2015

[3] Malavige GN, Fernando N, Ogg G (2011). Pathogenesis of dengue viral infections. Sri Lankan J Infect Dis.

[4] Kumar A, Balachandran V, Dominic A, Dinesh KR, Karim S, Rao G (2012). Serological evidence of leptospirosis and dengue coinfection in an endemic region in South India. Ann Trop Med Public Health.

[5] Levtt PN (2001). Leptspirosis. Clin Microbiol Rev.

[6] World Health Organization (2009) Dengue guidelines for diagnosis, treatment, prevention and control. WHO/HTM/NTD/DEN/2009.1. http://www.who.int/tdr/publications/documents/dengue-diagnosis.pdf. Accessed 9 Aug 2015 23762963

[7] Pappas G, Papadimitriou P, Siozopoulou V, Christou L, Akritidis N (2008). The globalization of leptospirosis: worldwide incidence trends. Int J Infect Dis.

[8] Reller ME, Bodinayake C, Nagahawatte A, Devasiri V, Kodikara-Arachichi W, Strouse JJ (2011). Leptospirosis as frequent cause of acute febrile illness in southern Sri Lanka. Emerg Infect Dis.

[9] Epidemiology Unit (2008). An interim analysis of leptospirosis outbreak in Sri Lanka-2008.

